# The earlier, the better: the effects of different administration timepoints of sorafenib in suppressing the carcinogenesis of VEGF in rats

**DOI:** 10.1007/s00280-017-3493-4

**Published:** 2017-12-01

**Authors:** Nan Li, Bin Chen, Run Lin, Ni Liu, Hai-tao Dai, Ke-yu Tang, Jian-yong Yang, Yong-hui Huang

**Affiliations:** 1 Department of Interventional Radiology, The First Affiliated Hospital of Sun Yat-sen University, 58th Zhongshan Road II, Guangzhou, 510080 People’s Republic of China; 2 The Central Hospital of Wuhan, Wuhan, People’s Republic of China

**Keywords:** Liver tumor, Vascular endothelial growth factor, Microvessel density, Sorafenib, Overall survival time

## Abstract

**Purpose:**

To investigate the optimal starting time point of sorafenib therapy in suppressing the tumor-promoting effects of VEGF up-regulation, which is frequently found after local therapy in clinical practice.

**Methods:**

VEGF was intravenously injected to imitate the evaluated expression after local tumor therapy, such as TACE. A total of 40 SD rats bearing hepatic tumors were randomly divided into four groups and sorafenib was administered at different timepoints: (A) control group: VEGF injection only; (B) initiating sorafenib 72 h prior to VEGF injection; (C) initiating sorafenib simultaneously with VEGF injection; (D) initiating sorafenib 72 h post-VEGF injection. The rate of tumor growth, median survival time, expression of VEGF, and microvessel density (MVD), as determined by immunohistochemical (IHC) examination, were compared.

**Results:**

The results revealed that the tumor size and median survival time were significantly different between the three sorafenib groups compared to the control group (*p* < 0.05). Median survival times were 19.6 ± 1.78, 31.2 ± 6.99, 27.4 ± 4.9, and 26.5 ± 4.6 days in group A, B, C, and D, respectively. Furthermore, there was a difference in statistical significance between the two sorafenib groups B and D (*p* = 0.04). Tumors were collected for HE staining and IHC examination. The expression levels of VEGF in B, C, and D were 42.8 ± 7.96, 71.9 ± 15.73, and 73.6 ± 13.73, and all of them were significantly lower than that in the control group (88.3 ± 13.61). Furthermore, the level of MVD was 109.2 ± 8.98 in the control group, which was significantly higher than in the three sorafenib groups (45.7 ± 16.92, 77.1 ± 16.29, and 93.6 ± 12.87, all *p* < 0.05).

**Conclusions:**

According to our results, the most suitable regimen for the administration of sorafenib is before the increased expression of VEGF, which showed a potential advantage for controlling the tumor growth and prolonging the survival time of test animal via inhibiting VEGF-receptor expression through the bifunction of VEGF, and the reduction of tumor angiogenesis.

## Introduction

Hepatocellular carcinoma (HCC) is the second most common cause of cancer-related death worldwide, and the liver is considered to be the most frequent site for blood-borne tumor metastases [1–3]. Local therapy, such as TACE, is considered to be an effective palliative treatment for patients with intermediate-advanced HCC [4–7]. These treatments only maintain the short-term stability of lesions, however, and provide a limited survival benefit [8]. Vascular endothelial growth factor (VEGF), one of the most potent factors mediating tumor angiogenesis, was markedly elevated in the majority of patients with HCC after local therapy and was considered to be one of the most important factors of tumor residue and recurrence [9, 10]. To date, the antiangiogenic multikinase inhibitor, sorafenib, blocks the activity of the Raf serine/threoninekinase and receptor tyrosine kinases, such as VEGFR-2, PDGFR-β, c-KIT, FLT-3, and RET, resulting in the inhibition of tumor proliferation and neovascularization [11]. The efficacy of sorafenib alone in BCLC stage C HCC has been proven [12–14]. However, its partial response was 3.3% and drug resistance developed in 6.5 months in patients treated with sorafenib [13]. Furthermore, it is not comprehensive enough for BCLC staging in describing the patients’ condition that BCLC stage C includes patients with portal vein tumor thrombus (PVTT), while trunk or branch PVTT is not distinguished clearly in this stage. Safety and efficacy of TACE on HCC with portal vein invasion have been proven [15]. Even though sorafenib treatment significantly increased median overall survival (OS) in BCLC B stage, it is not recommended to administer sorafenib in such stage accoding to current guidelines [4, 16]. In fact, there are no clear taboos for the two kinds of treatments to most patients with stage B and C. Combined therapy, such as TACE + sorafenib, bears hope for better prognosis. Retrospective studies showed that the combination of TACE + sorafenib could be beneficial for patients with intermediate/advanced HCC [17]. A recent study suggested that this combination might be indicated for patients with BCLC stage B HCC [18], but a phase III trial suggested that this approach should only be used in selected patients [13]. Strebel et al. [19] have proposed three models for the combination of TACE + sorafenib: the sequential, interrupted, and continuous models. Meanwhile, we found that different studies have adopted different models and administration times of sorafenib for patients undergoing TACE [20, 21]. Apparently, these differences may lead to controversies across these trials [21]. In this study, an animal experiment was performed to investigate the optimal starting time point of sorafenib administration in the combined therapy.

## Materials and methods

### Tumor cells and animals

Walker-256 tumor cells were obtained from the Cell Bank of Sun Yat-sen University Experimental Animal Center and all animals were obtained from the Experimental Animal Center of Sun Yat-sen University. 4-week-old BALB/c nude mice (body weight, 12–15 g; *n* = 2) received injections of tumor cells at a site on the lateral thigh; 5- to 7-week-old male Sprague–Dawley (SD) rats (body weight, 150–200 g; *n* = 50) were used for intrahepatic tumor inoculation. Animals were housed under specific pathogen-free conditions with an air-conditioned animal cage at a temperature of 23 ± 3 °C and a relative humidity of 50 ± 10%. All experimental protocols involving animals were approved by the animal ethics committee of Sun Yat-sen University.

### Establishment of the subcutaneous tumor model

The Walker 256 carcinosarcoma cells used for the establishment of the liver tumor models were thawed from − 80 °C and harvested as previously described [22, 23]. The tumor cell suspension was then diluted to 10^7^ cells/mL with phosphate-buffered saline (PBS, 16.5 mM phosphate, 137 mM NaCl, and 2.7 mM KCl, at pH 7.4) and the mixture was subcutaneously inoculated into BALB/c nude mice at a site on the lateral thigh. About 9 days after tumor cell inoculation, subcutaneous tumors of approximate 1 cm in diameter could be detected at the injection site.

### Establishment of the hepatic cancer model

Tumor implantation was performed using a previously described method [24–26]. After mincing the subcutaneous tumors from a donor animal into small cubes of about 1.0 mm^3^, SD rats were laparotomized through a midline abdominal incision under intraperitoneal anesthesia with 10% chloral hydrate at a dosage of 300 mg/kg. The left lateral lobe of the liver was allowed to protrude out of the abdominal cavity and a subcapsular tunnel of a depth about two-third of the lobe thickness was made with ophthalmic tweezers. A solid tumor tissue cube was then inserted into the tunnel and the wound was covered with a small piece of gelfoam (Jinling Pharmaceutical, Nanjing, China). Hemostasis was not a necessity. All experimental rats were subsequently returned to their cages to recover after surgery and regain normal activity the next day.

### Experimental treatment

Ten days after implantation, magnetic resonance imaging (MRI) was used to evaluate the establishment of the liver tumor model. The experimental animals were anesthetized with intraperitoneal injection of chloral hydrate and were then placed on an MRI micro-coil and scanned (Magnetom Avanto 3.0 T; Siemens, Washington, DC) with both T_1_WI and T_2_WI scanning sequences. The scanning parameters were as follows: echo times of 82.0 ms for T_1_WI and 1.5 ms for T_2_WI; repetition times of 1500 ms for T_1_WI and 508.7 ms for T_2_WI; reconstructed slice thickness of 5 mm for both T_1_WI and T_2_WI.

Forty SD rats were eligible with liver tumor from the 50 rats and were randomly divided into four groups: Group A (A, *n* = 10) was control group in which the animals received VEGF (20 mg/L, 1 ml/kg, body weight) [27] injection only. Group B (B, *n* = 10) was given a gavage of sorafenib (100 mg/kg, body weight) [28] 72 h prior to the injection of VEGF. Group C (C, *n* = 10) was given a gavage of sorafenib simultaneously with the VEGF injection. Group D (D, *n* = 10) was given a gavage of sorafenib 72 h after the VEGF injection. Sorafenib was administered p.o. once daily for 10 days at a dose levels of 100 mg/kg body weight. Recombinant rat VEGF165 (20 mg per tube; PeproTech, Rocky Hill, NJ) was centrifuged for 30 s at 1000 rpm, dissolved in 100 mL of deionized water, and diluted with a normal saline solution to the desired concentration. Rats in groups B, C, and D received one injection of 20 mg/L VEGF.

### Imaging and pathological investigations

MRI was used to evaluate the development of tumors and ascites [4, 29]. It was performed to ensure the liver implantation of tumor tissue and to monitor the development of the tumors and ascites 10 days after the administration of sorafenib.

In case of rapid tumor progression, the general condition of the animals, which was defined in terms of the condition of the coat, nutrition intake, and behavior, was assessed daily. Liver tissues were used to analyze and compare the MVD and VEGF levels of rat liver tumor biopsies, and were obtained after the animals died naturally. We used the Leica Microsystems, the rabbit anti-rat VEGF-165 polyclonal antibody (BOSTER, Inc, Wuhan, China), and rabbit anti-rat CD34 polyclonal antibody (BOSTER, Inc, Wuhan, China). Briefly, 4 mm serial histological sections from formalin-fixed paraffin embedded blocks of tumor tissue were dewaxed in xylene, rehydrated through graded alcohols, immersed in 10 mM Tris and 0.5 M EDTA at pH 9.0, and were finally microwaved twice for 5 min each. Subsequently, the sections were incubated with 3% H_2_O_2_ for 10 min to block endogenous peroxidase activity. The sections were then incubated overnight at 4 °C with the primary antibodies (dilutions: VEGF2, 1:40; CD34, 1:40).

### VEGF and microvessel detection and counting

The level of VEGF protein expression and MVD was independently determined by two senior pathologists blinded to every slide. The number of epithelial cells that exhibited positive cytoplasmic immunoreactivity to VEGF was determined by counting 100 epithelial cells in every slide and five random sights in every section sample were selected to count stained epithelial cells. The tumor vasculature was examined using an average amount of CD34-positive microvessels. Samples were examined by light microscopy and five areas with the highest numbers of stained microvessels were identified as ‘hot spots’. For the quantitation of microvessels, we counted each of the five ‘hot spots’ using a 200× magnification field and took the average. Any cell or cell cluster showing positive CD34 staining was counted as a vessel, as described in the Weidner method [10, 30]. No counts were performed in areas of necrosis or inflammation. Sections, for which five ‘hot spots’ could not be identified, were excluded from further analysis. If two pathologists had significant differences, sections were reviewed again until they reached a consensus.

### Statistical analysis

SPSS 20.0 was used for all analysis. Data are presented as mean ± SD, and VEGF and MVD comparisons were performed using repeated-measures analysis of variance with Student–Newman–Keuls for the post hoc test. Kaplan–Meier curves were utilized for median survival time, and intergroup comparisons were made using log-rank tests. Spearman’s rank correlation coefficients were computed to estimate the correlations of median survival time and VEGF and MVD expression. A *p* value < 0.05 was set as the cutoff for statistical significance.

## Results

### Growth of detectable tumors in liver

**Fig. 1:**
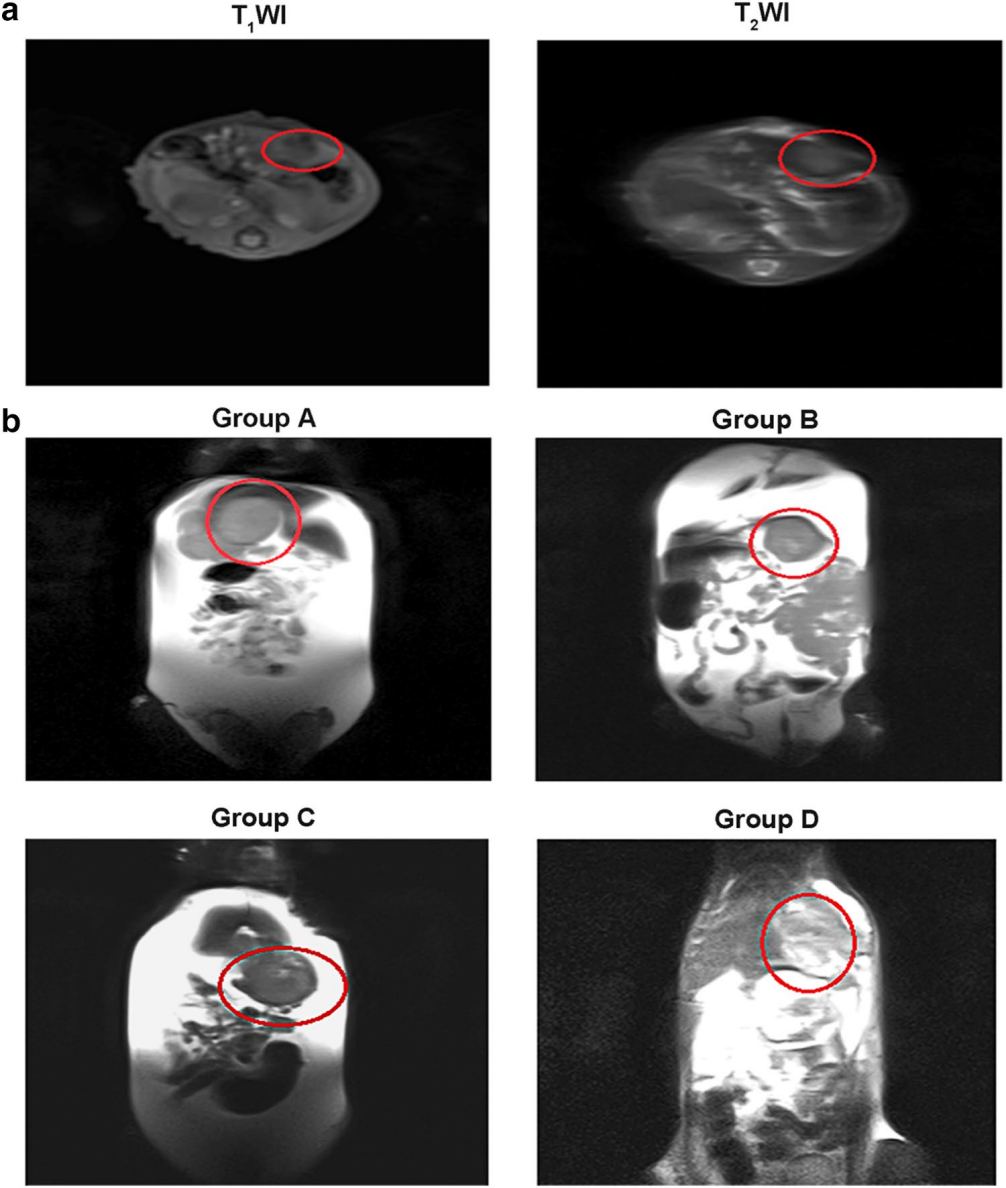
**a** MRI of a rat liver 10 days after tumor tissue implantation. The tumor (red circle) is evident as a slightly hypointense mass in T_1_WI and a slightly hyperintense mass in T_2_WI. The size of the tumor was relatively small. **b** T_2_WI MRI coronal scans of rats livers from each of the four experimental groups, 10 days after administrating sorifenib. The tumor (red circle) is evident as a slightly hyperintense mass, within which the necrosis area shows a higher signal. The tumors in the liver did not differ significantly in size or morphology among the sorafenib groups at this time point. The hyperintense region in the abdomen suggested significant ascites. *MRI* magnetic resonance imaging, *VEGF* vascular endothelial growth factor

10 days later after the implantation of tumor tissues, MR routine scanning was utilized to confirm whether or not there was a liver tumor in the SD rat liver (Fig. 1a). The size and location of tumors were recorded from images of decent quality. Detectable tumors, excluding those in the abdominal wall or abdominal cavity, were accepted for the following experiments. 10 days after administration of sorafenib, we used MR scanning to measure tumor size (Fig. 1b; Table 1). The maximum diameter of the tumor was used as an evaluation index.

**Table 1:** Changes in tumor size, as measured by MRI

Rat no.	A		B		C		D	
	10 days	20 days	10 days	20 days	10 days	20 days	10 days	20 days
1	1.4	5.4	2.0	5.2	1.9	5.0	1.8	4.9
2	1.2	--	1.8	4.9	1.7	4.2	1.5	4.6
3	1.9	--	1.5	4.6	1.9	4.9	2.1	5.0
4	1.8	--	1.7	4.7	1.8	--	1.9	5.3
5	1.2	--	1.4	4.2	2.0	4.9	1.6	4.8
6	2.0	5.7	1.9	5.4	1.6	4.4	2.0	5.1
7	1.7	5.6	1.8	4.6	1.9	4.8	1.8	4.5
8	2.1	--	2.2	5.8	2.1	5.1	1.9	4.7
9	1.9	--	2.0	4.1	2.0	5.2	2.1	--
10	1.6	4.9	1.5	4.3	1.7	4.7	1.5	4.1
Mean ± SD	1.68 ± 0.32	5.40 ± 0.36	1.78 ± 0.26	4.78 ± 0.55*	1.86 ± 0.16	4.80 ± 0.35*	1.82 ± 0.23	4.78 ± 0.36*

Tumor size is given in cm

*p < 0.05 vs. control group

-- death, *MRI* magnetic resonance imaging, *SD* standard deviation, *A* control group: VEGF injection only, *B* initiating sorafenib 72 h prior to VEGF injection, *C* initiating sorafenib simultaneously with VEGF injection, *D* initiating sorafenib 72 h post-VEGF injection

### Medians for survival time

**Fig. 2:**
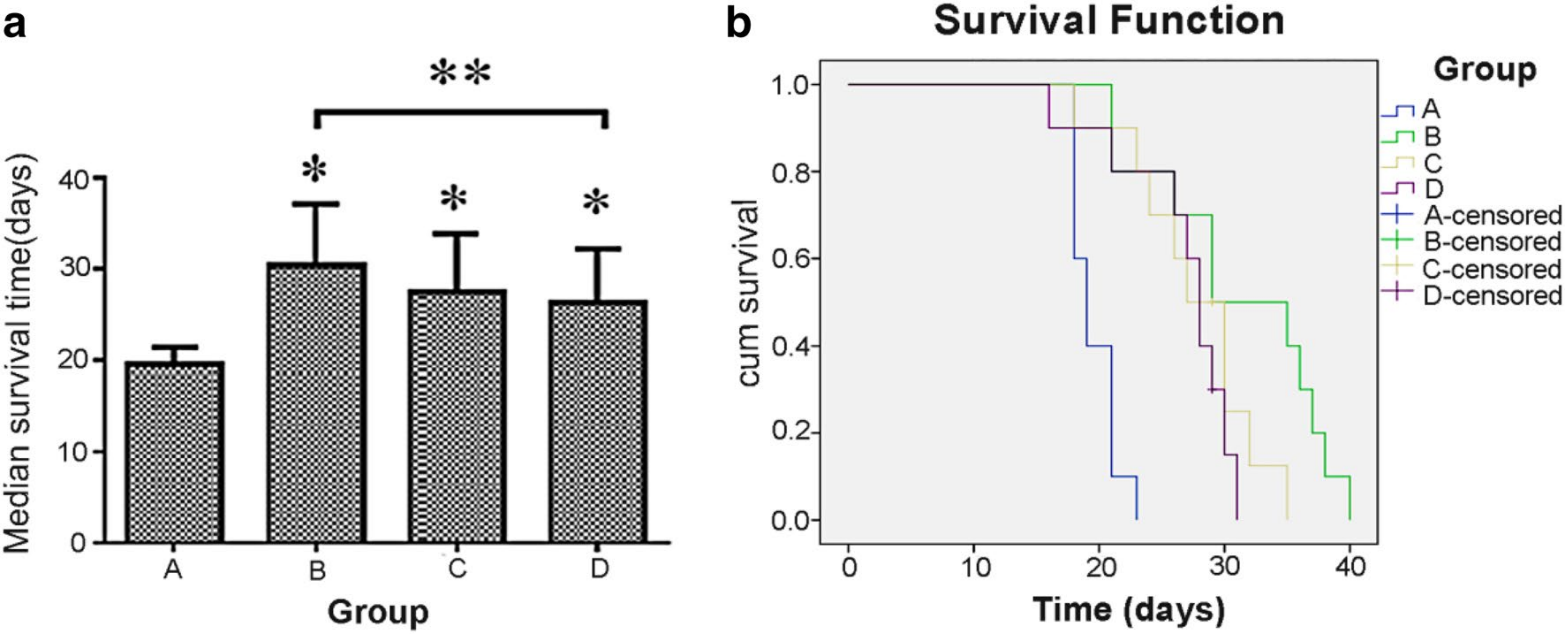
**a** Median survival time for the animals from all four groups. (*p < 0.01 vs. A, **p < 0.05 between two groups shown by horizontal line). **b** Survival curves for the rats in the four experimental groups. The median survival was 19.6 days in the control group (blue curve), 31.2 days in group B (green curve), 27.4 days in group C (yellow curve), and 26.5 days in group D (brown curve). The log-rank test revealed significant differences between survival time in the control and all the sorafenib groups (*p* < 0.01)

The median survival times of different groups were 19.6 ± 1.78, 31.2 ± 6.99, 27.4 ± 4.9, and 26.5 ± 4.6 days in groups A, B, C, and D, respectively (Fig. 2a, b). The results revealed that the survival times were significantly different between groups subjected to sorafenib and the control (*p* < 0.01). Significant differences were also found between groups B and group D (*p* = 0.04). However, groups B and C (*p* = 0.09) and groups C and D (*p* = 0.69) had no statistic differences (Fig. 2a).

### Pathological examination

**Fig. 3:**
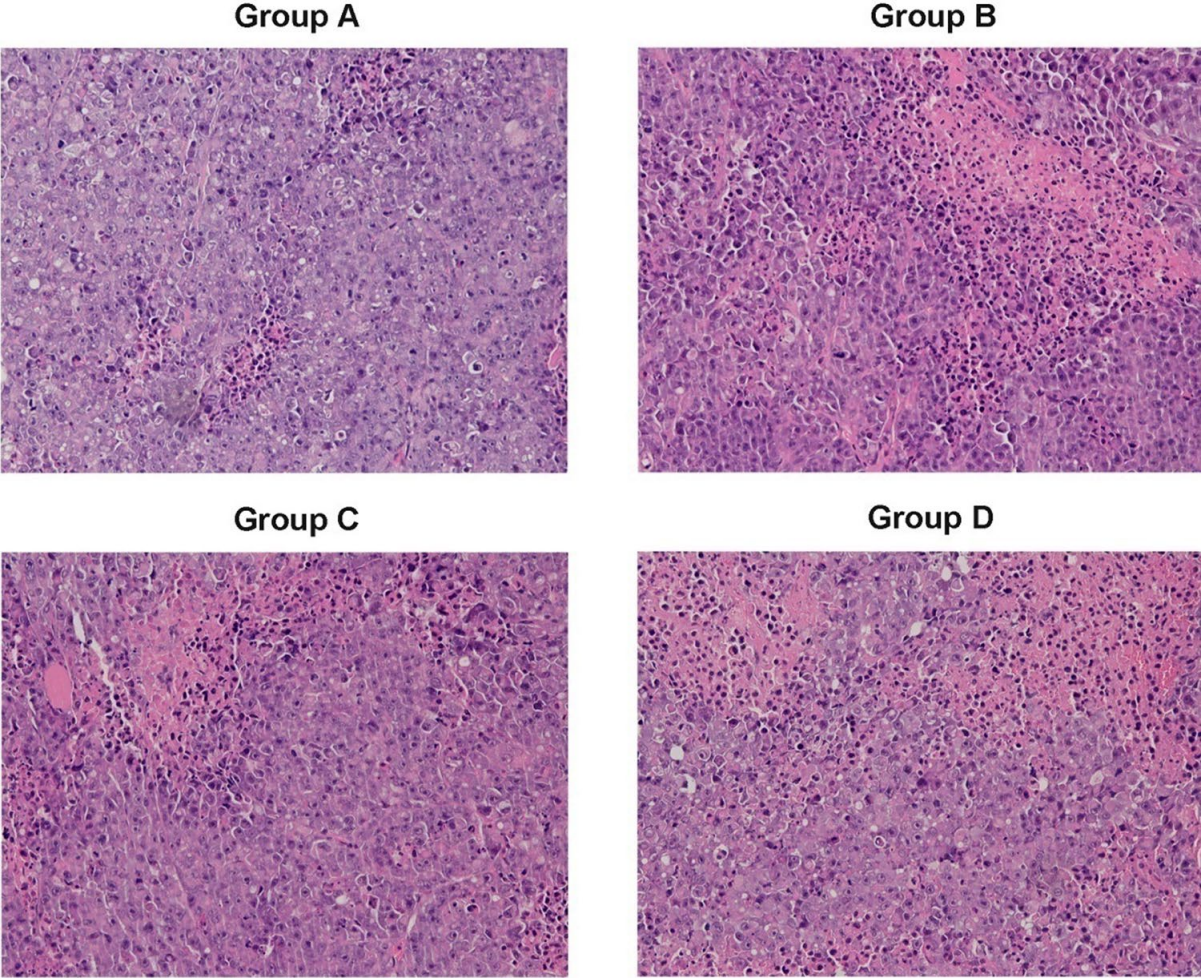
(HE staining; magnification, 200) Group A: VEGF injection only; Group B: initiating sorafenib 72 h prior to VEGF injection; Group C: initiating sorafenib simultaneously with VEGF injection; Group D: initiating sorafenib 72 h post-VEGF injection. Representative histochemical images showing the cancer morphology of rats with hepatic metastases. Tumor cells with significant atypia are evident and the eosinophilic area shows dead cells

After the natural death of the experimental animals, we collected the liver tumor specimens to perform HE staining (Fig. 3). As shown in the pictures, the normal lobular architecture disappeared and tumor cells were arranged as sheets or nests. The tumor cell nuclei had a high frequency of mitoses. Tumor atypical cells were evident, such as dual- and even multi-core. A large eosinophilic patch in the visible area was a tumor necrosis area. The tumors did not differ significantly in morphology between the four groups. However, compared with the sorafenib groups, the tumor cells in the control group were smaller and crowded and varied more significantly both in size and shape.

### Immunohistochemical methods

**Fig. 4:**
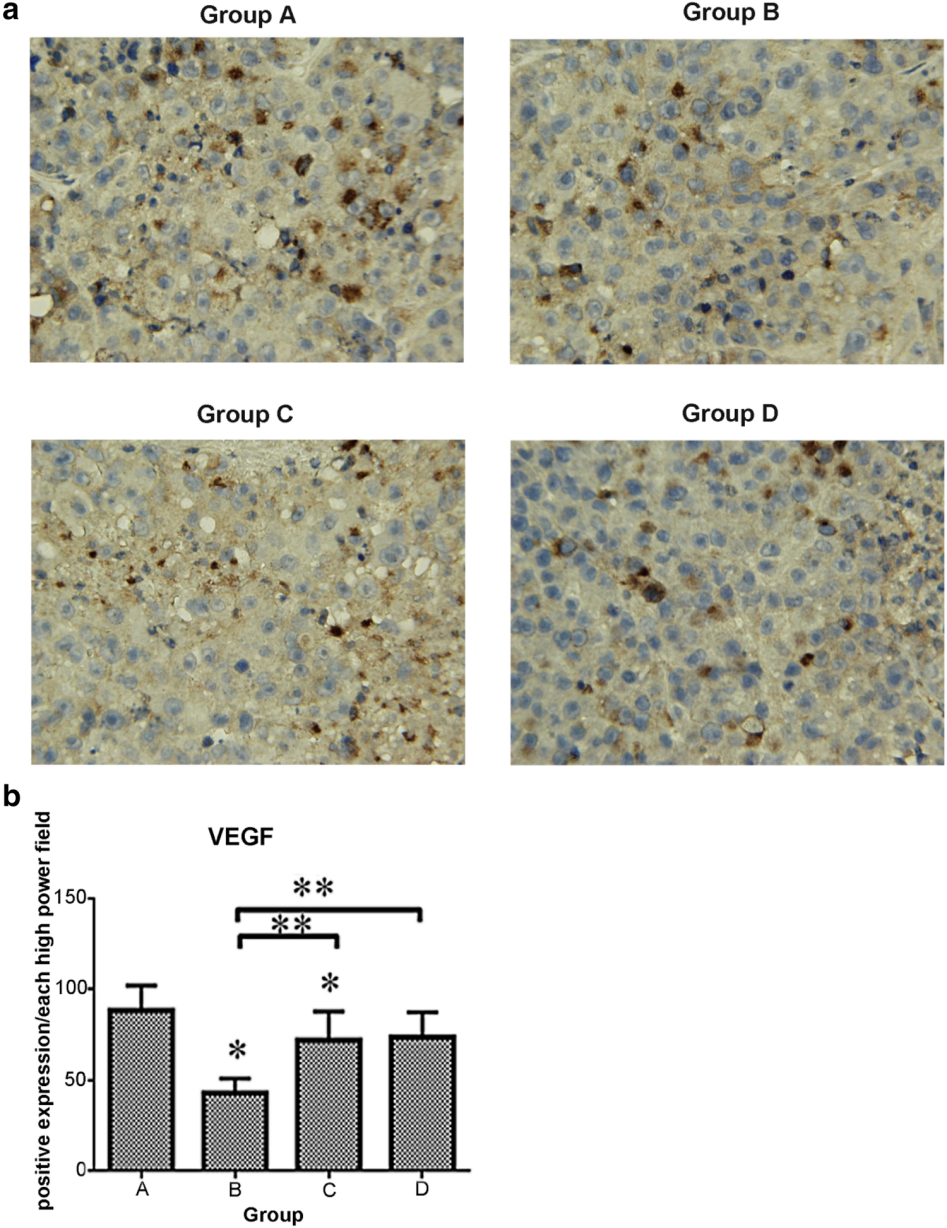
**a** (VEGF, magnification, 400) IHC analysis of VEGF expression in tumor tissues. Group A: VEGF injection only; Group B: initiating sorafenib 72 h prior to VEGF injection; Group C: initiating sorafenib simultaneously with VEGF injection; Group D: initiating sorafenib 72 h post-VEGF injection. Positivity for VEGF was identified by dark-brown staining VEGF expression in vascular endothelial cells was high in the control group and lower in the sorafenib groups (A vs. B *p* < 0.001, A vs. C *p* = 0.008, A vs. D *p* = 0.017). **b** Expressions of VEGF in the four groups (*p < 0.05 vs. A. **p < 0.05 between the two groups shown by horizontal line). *VEGF* vascular endothelial growth factor

**Fig. 5:**
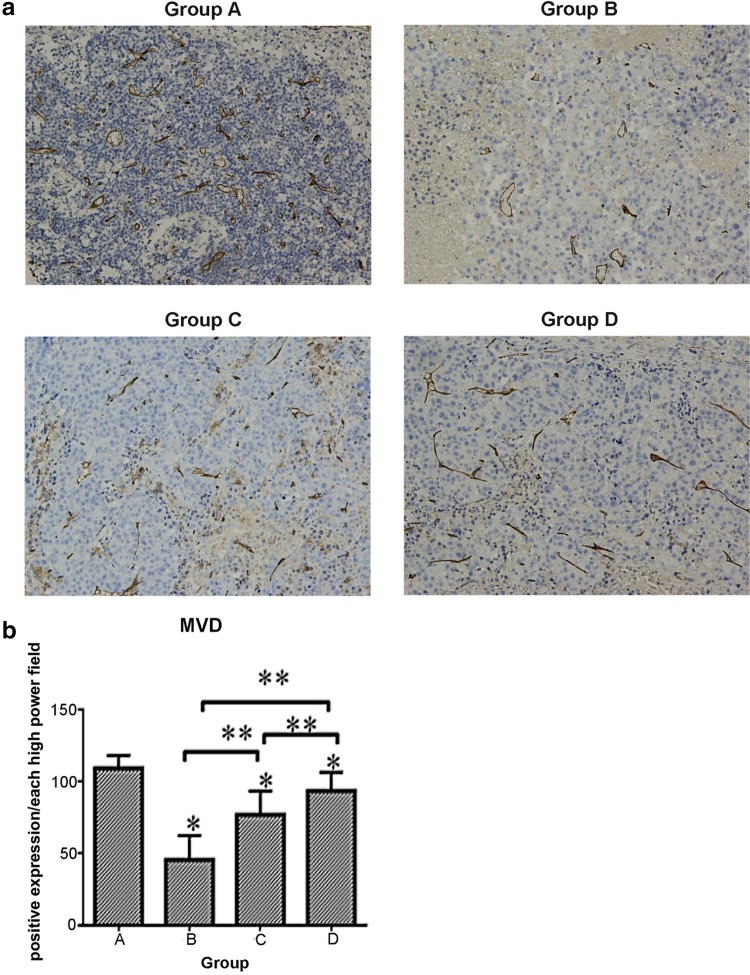
**a** (MVD, magnification, 200) IHC analysis for MVD expression in HCC tissue. Group A: VEGF injection only; Group B: initiating sorafenib 72 h prior to VEGF injection; Group C: initiating sorafenib simultaneously with VEGF injection; Group D: initiating sorafenib 72 h post-VEGF injection. Positive CD34 cells were identified by brown staining. The abundance of VEGF-positive tumor cells in the control group is clearly higher than that in the experimental group (*p* < 0.01). **b** Expressions of VEGF in the four groups (*p < 0.05 vs. A. **p < 0.05 between the two groups shown by horizontal line)

Hepatic cancer VEGF expression appeared as brown staining that was, for the most part, mainly diffusely distributed in the cytoplasm. In the control group, a huge number of VEGF-positive vascular endothelial cells were detected with immunohistochemistry (IHC) (Fig. 4a). The number of samples containing VEGF-positive cells in the control group, 88.3 ± 13.61, was higher than those of the sorafenib groups (*p* < 0.05), 42.8 ± 7.96, 71.9 ± 15.73, and 73.6 ± 13.73 in groups B, C, and D, respectively. VEGF was also expressed in a stepwise regression from group B to group D (Fig. 4b), demonstrating that the earlier the feeding time, the more apparent the VEGF inhibition effect of VEGF and the greater the influence on the tumor sorifenib had. CD34-positive cells appeared as brown or dark-brown staining (Fig. 5a). The trend of MVD was just like that of VEGF in the four groups (109.2 ± 8.98, 45.7 ± 16.92, 77.1 ± 16.29, and 93.6 ± 12.87, respectively). Statistical differences did exist between the control group and sorafenib groups (*p* < 0.01) (Fig. 5b).

### Associations between median survival time and neoangiogenesis

VEGF and MVD are commonly utilized to assess the angiogenic activity of tumors: they are consistently associated and their correlational coefficient was 0.875, which was positive. Using Pearson’s correlation analysis of continuous random variables, the Pearson’s coefficient product-moment correlation of VEGF and median survival time was 0.78, which was negative. The correlation between MVD and median survival time was also negative at 0.794.

## Discussion

HCC is a cancer with a devastating prognosis [31]. Local therapy, especially TACE, is a preferred palliative treatment for unresectable HCC and has been proven to prolong overall survival based on high-level evidences [32, 33]. However, these treatments only maintain the stability of the lesion for a short time and provide a limited survival benefit [8]. The pitfalls of local therapy in HCC are tumor residue and recurrence [32], in which sustained angiogenesis is considered to play a critical role [34–36]. Vascular endothelial growth factor (VEGF), one of the most potent factors mediating tumor angiogenesis [37], was markedly elevated in the majority of patients with HCC after local therapy [9, 10]. In hypervascular tumors characterized by substantial neovascularization [38], high VEGF expression correlates to more malignant biological behavior, including infiltration, recurrence, and metastasis [37]. Among local therapy, TACE is one of the vasculo-occlusive and hypoxic challenges to tumor, and VEGF has more obvious impact on recurrence of residual tumor [39]. A connection has been shown between HIF-1α levels and VEGF concentration after TACE treatment, and HIF-1α can increase the liver tumor blood reperfusion up-regulation of VEGF level, thereby leading to the blood reperfusion in residual liver tumor resulting in bad prognosis [34, 35, 40]. In our previous study, we established an orthotopic liver cancer model and determined the dose–response relationship between VEGF and tumorigenesis. Our preliminary experiment proved that exogenous VEGF has an obvious growth promotion effect on liver tumors and that the safest dose is 20 mg/L [27]. Microvescular density (MVD) is commonly used to assess the angiogenic activity of tumors. MVD is determined by counting CD34-positive vessels, since CD-34 is a vascular endothelial cell proliferation marker and only expressed in the vascular endothelial cells of tumor tissues and not in the vessels of healthy tissues [41]. This characteristic could be used to simulate the effect of VEGF on residual tumor after local therapy.

Sorafenib, as a multikinase inhibitor with antiangiogenic and antiproliferative properties, is the only systemic therapy shown to confer a survival advantage in patients with unresectable advanced HCC [12, 13, 42]. In the pivotal Sorafenib Hepatocellular Carcinoma Assessment Randomized Protocol (SHARP) trial and other randomized controlled trials for patients with advanced HCC, sorafenib treatment significantly increased median overall survival (OS) [13, 43]. Nevertheless, in the current guidelines, sorafenib is still only recommended for stage C HCC in BCLC and other algorithms [4]. Pawlik et al. [17] found that TACE + sorafenib could improve the prognosis of HCC. Conversely, Kudo, et al. [20] had reported that sorafenib conferred no added benefit. We noticed that differences in timings of antiangiogenic treatment for patients undergoing TACE across these trials were suspected as one important factor of the controversies. Based on our experience, we executed an animal experiment to investigate the effects of sorafenib on liver cancer with VEGF overexpression, and to ascertain valuable information that will improve combination treatments of TACE and sorafenib.

Some literatures have suggested that sorefenib may have a bifunction in suppressing the effects of VEGF and reducing tumor angiogenesis, resulting in increased ischemic hypoxia in the tumor tissue and slowed growth [44]. In this study, the results of MR scans revealed that tumor growth was significantly suppressed in groups treated with sorafenib compared to the control group (*p* < 0.01), which had confirmed the viewpoint. Meanwhile, we harvested hepatic tumor tissues from animals that had died naturally and performed HE staining and IHC. The expressions of VEGF in the four groups were 88.3 ± 13.61, 42.8 ± 7.96, 71.9 ± 15.73, and 73.6 ± 13.73, and those of MVD were 109.2 ± 8.98, 45.7 ± 16.92, 77.1 ± 16.29, and 93.6 ± 12.87. The correlation coefficient of Pearson’s product-moment correlation between MVD and VEGF was plus 0.875. This was consistent with the results of Dufour et al. [45] who demonstrated that sorafenib could reduce the concentration of plasma VEGF from 93 to 67 ng/L post-TACE. Our findings, demonstrating that sorafenib may effectively reduce tumor-induced angiogenesis, was also verified by HE staining. Compared with the control group, the HE staining pictures of the treated groups showed more eosinophilic areas without obvious cell structure under a light microscope, which means that a large number of tumor cells were dead because of the ischemic hypoxia and necrosis. The better prognosis of animals receiving sorafenib will be divinable on account of tumor cell death. The survival times of the four groups were 19.6 ± 1.78, 31.2 ± 6.99, 27.4 ± 4.9, and 26.5 ± 4.6 days, respectively, showing that the animals in the sorafenib groups lived longer than those in the control (*p* < 0.01). Although no statistical significance existed between the sorafenib groups, the survival times showed a decreasing trend that corresponded with the administration time of sorafenib. These results presented evidence that the earlier an animal receives sorafenib, the greater the inhibition of tumor angiogenesis. It could be explained as sorafenib can more effectively decrease the expression of VEGF before it binds with the VEGF receptor; once VEGF has bound with the receptor and the chain reactions is initiated, the function of sorafenib will be weakened.

In conclusion, the best regimen of sorafenib administration was administering sorafenib before the increased expression of VEGF, which could confer the greatest survival benefit by inhibiting VEGF-receptor expression via the bifunction of VEGF, and reducing tumor angiogenesis.
